# Communication between healthcare providers and medical cannabis patients regarding referral and medication substitution

**DOI:** 10.1186/s42238-021-00058-0

**Published:** 2021-01-24

**Authors:** Kevin F. Boehnke, Evangelos Litinas, Brianna Worthing, Lisa Conine, Daniel J. Kruger

**Affiliations:** 1 Anesthesiology Department, University of Michigan Medical School, 24 Frank Lloyd Wright Drive, Ann Arbor, MI 48106 USA; 2 Ann Arbor, USA; 3 Population Studies Center, University of Michigan, Ann Arbor, MI USA

**Keywords:** Medical cannabis, Medication substitution, Healthcare provider knowledge, Medical cannabis referral

## Abstract

**Background:**

People report using cannabis as a substitute for prescription medications but may be doing so without the knowledge of their primary health care providers (PCPs). This lack of integration creates serious concerns, e.g., using cannabis to treat medical conditions that have established treatment options.

**Methods:**

We conducted an anonymous, cross-sectional online survey among patrons of a medical cannabis dispensary in Michigan (*n* = 275) to examine aspects of their relationship with their PCP and their perceptions of PCP knowledge related to cannabis.

**Results:**

Overall, 64% of participants initiated medical cannabis use based on their own experiences vs. 24% citing advice from their PCP. Although 80% reported that their PCP knew they currently used medical cannabis, 41% reported that their PCP had not always known. Only 14% obtained their medical cannabis authorization from their PCP. Only 18% of participants rated their PCP’s knowledge about medical cannabis as very good or excellent and only 21% were very or completely confident in their PCP’s ability to integrate medical cannabis into their treatment. Although 86% had substituted cannabis for pharmaceutical medications, 69% (*n* = 134) of those who substituted reported some gap in their PCP’s knowledge of their substitution, and 44% (*n* = 86) reported that their PCP was currently unaware of their substitution.

**Conclusions:**

Patients frequently substitute cannabis for prescription drugs, often without PCP knowledge. Although most participants disclosed cannabis use to their PCP, their perceptions of PCP knowledge ranged widely and many obtained medical cannabis licensure from an outside physician. Our results highlight the need for standardized physician education around appropriate medical cannabis use.

**Supplementary Information:**

The online version contains supplementary material available at 10.1186/s42238-021-00058-0.

## Introduction

Thirty-five states in the USA have enacted medical cannabis programs. Despite being designated a Schedule I drug under the 1970 Controlled Substances Act in the US (indicating a high potential for abuse and no accepted therapeutic use), a recent National Academies of Sciences, Engineering, and Medicine report found evidence supporting the therapeutic value of cannabinoids (active compounds in cannabis) for chemotherapy induced nausea and vomiting, chronic pain, and multiple sclerosis-related spasticity (National Academies of Sciences, Engineering, and Medicine 2017). However, the evidence for most conditions allowed by state medical laws (e.g., depression) was insufficient (National Academies of Sciences E, and Medicine 2017; Boehnke et al. 2019a). Complicating this mismatch are scientific and news reports of individuals substituting cannabis for opioids and other prescription medications (Boehnke et al. 2016; Boehnke et al. 2019b; Lucas et al. 2016; Lucas and Walsh 2017; Lucas et al. 2019; Reiman et al. 2017; Piper et al. 2017; Corroon Jr. et al. 2017; Rod 2019)—including for conditions for which there is limited evidence that cannabis has therapeutic value (e.g., anxiety). Similarly, many individuals using cannabis believe that cannabis is useful for medical conditions with no evidence base (e.g., cancer treatment) (Kruger et al. 2020). Taken together, these findings highlight the need for a strong healthcare provider presence in conversations about safe cannabis use in the context of medication substitution.

Whether this substitution occurs with oversight from healthcare providers remains unknown, but healthcare providers consistently express a lack of knowledge about medical cannabis, demonstrated by studies showing that only 9% of medical schools cover medical cannabis (Evanoff et al. 2017) and ~ 80% of physicians reported needing additional cannabis education (Kondrad and Reid 2013). Further, when physicians are approached for medical cannabis recommendations, there are no formal guidelines for appropriate medical use. Among patients, those using cannabis may not approach healthcare providers for fear of stigma or legal trouble. Indeed, some institutional policies prevent physicians from recommending medical cannabis (Carlini et al. 2017), and patients may lose employment due to a positive drug screen even if they have a medical cannabis license (Kulig 2017). As such, many people may use cannabis without the knowledge of or input from their primary healthcare providers (PCPs) (Kruger et al. 2020), emphasizing the lack of integration of medical cannabis into mainstream healthcare settings.

In the current study, we further explored this lack of integration by surveying individuals using medical cannabis in Michigan, where cannabis is legal for medical and adult use (since 2009 and 2018, respectively). We hypothesized that although many participants would report substituting cannabis for medications, most would do so without PCP guidance. We also hypothesized that participants would report low PCP comfort and knowledge regarding medical cannabis.

## Methods

### Setting and participants

We invited patrons of Om of Medicine—a medical cannabis dispensary in Ann Arbor, Michigan—to complete an anonymous, online survey (administered via Qualtrics) through flyers, emails, and social media between April 2019 and February 2020. Emails with the survey link as well as other information (e.g., product specials) were sent out each month to the client database (~ 5000 people) approximately once per month, and flyers were located around the facility. Social media notices were made publicly without additional advertising. Dispensary staff informed patrons about the research study but otherwise had no involvement with their participation, and no special privileges or attention were given to individuals who chose to participate in the research. In Michigan, patient registry licenses are valid for two years and individuals can obtain licensure both from their PCP or from an outside provider who must be an MD or DO licensed in Michigan (Marijuana Regulatory Agency LaRA 2020).

Participants were > 18 years old and currently used cannabis for medical purposes. Participants answered questions on demographic information (sex, ethnicity, age, education), medical cannabis use and related substitution behaviors, and healthcare provider knowledge and attitudes toward medical cannabis. All procedures and surveys were approved as an exempt study by the Institutional Review Board at the University of Michigan under protocol HUM00165859. Participants freely consented to participate and were not compensated. Most respondents completed the survey (*n* = 275), 30 cases with incomplete data were not included.

### Measures

Measures were adapted from several other studies of medical cannabis use and cannabis substitution (Boehnke et al. 2019b; Lucas and Walsh 2017; Kruger et al. 2020; Kruger and Kruger 2019).

#### Reasons for cannabis use

Participants selected their primary condition for using medical cannabis from an extensive list of options which we have used in other surveys (Kruger et al. 2020; Kruger and Kruger 2019). Participants indicated why they used medical cannabis from the following list of reasons: *my own experiences*; *advice from my primary health/medical care provider*; *advice from my medical marijuana caregiver/dispensary*; *advice from other individual(s)*; and *other source of information* (Kruger et al. 2020; Kruger and Kruger 2019).

#### Patient disclosure of medical cannabis use to PCP

Participants were asked: “Does your primary care provider (PCP) know that you use medical marijuana?” and “Are you seeing (or did you see) your PCP for the health issue that you use medical marijuana to help treat?” Participants whose PCPs knew about their use were asked: “How did your PCP find out that you use medical marijuana?” and “Was there a time when your PCP did not know that you used medical marijuana?” Participants whose PCPs did not know about their use were asked: “Did your PCP ever ask you about medical marijuana?” and “Is there a reason why you did not tell your PCP about your medical marijuana use?”, with an open-ended text box to explain. Reported reasons were categorized by reoccurring themes.

#### Medical cannabis license authorization

Participants were asked whether their PCP authorized their cannabis license, the number of doctors visited to obtain their license, and whether their PCP was in contact with the physician who authorized their license. Those whose PCP did not authorize their license were asked how they found their authorizing physician, with response options: referred by my PCP; referred by a friend or family member; in a newspaper (Metrotimes, etc.); Internet search; and other: with an open-ended text box. These participants were also asked whether the doctor authorizing the license is still involved in their health care, and if they ever saw the authorizing physician again.

#### Perceptions of PCP knowledge and support for medical cannabis

Participants rated their PCP’s knowledge of medical cannabis as *poor*, *fair*, *good*, *very good*, and *excellent*; confidence in their PCP’s ability to integrate medical cannabis into their treatment as *not at all confident*, *somewhat confident*, *moderately confident*, *very confident*, and *completely confident*; and perceptions of their PCP’s support of medical cannabis: *not at all supportive*, *somewhat supportive*, *moderately supportive*, *very supportive*, and *completely supportive.* All 5-point Likert-type scales were converted to continuous values (1–5) for statistical analyses.

#### Substitution measures

Participants were asked, “Have you ever or are you currently using or taking...” with response options for a wide range of drug classes used in other studies (Kruger and Kruger 2019). Participants who indicated using a drug class were asked, “Have you reduced your use of or stopped using [drug] because of medical marijuana?” (Boehnke et al. 2019b). Those who responded affirmatively selected the reasons why from the following: my own experiences; advice from my primary health/medical care provider; advice from my medical marijuana caregiver/dispensary; advice from other individual(s); and other source of information. These participants were also asked “Did your PCP know that you reduced or stopped your use of [drug] because of medical marijuana?” with response options of “Yes, immediately”, “Yes, but not immediately”, and “No”.

### Statistical analyses

Descriptive analyses included frequencies and mean scores, with selections of subgroups as appropriate. Independent samples t-tests compared perceptions of PCP knowledge about medical cannabis, confidence in PCP’s ability to integrate medical cannabis into their treatment, and perceived PCP level of support of medical cannabis by whether or not their PCP knew that they used medical cannabis; whether their PCP had delayed knowledge of their medical cannabis use; whether their PCP knew that they substituted medical cannabis for pharmaceutical drugs; whether their PCP had delayed knowledge of their substitution, and whether their PCP had authorized their medical cannabis card. Chi-square analyses tested whether the distribution of participants whose PCP had authorized their medical cannabis license (compared to those who had not) was different with regards to gaps in knowledge of substitution and whether participants substituted cannabis for pharmaceutical drugs. All analyses were conducted in SPSS (IBM, Armonk, NY).

## Results

### Reasons for use and sociodemographics

Participants (*N* = 275, see Table 1 for demographics) used cannabis to treat various issues, primarily chronic pain (31%), PTSD (7%), arthritis (6%), headaches/migraines (6%), anxiety/panic attacks (5%), cancer/leukemia (5%), depression (4%), and stress (4%). These findings are somewhat consistent with registration patterns in Michigan: during the time of this survey, “chronic pain” or “severe and chronic pain” were the most common qualifying conditions in the state (52.2% and 54.6% of conditions), followed by arthritis (20.2%), cancer (5%), and PTSD (4.7%) (Agency MMR 2019). Depression, anxiety, and stress are common comorbidities of chronic pain (Schrepf et al. 2018), and many patients report using cannabis for both pain and mental health symptoms (Lucas et al. 2019). Most participants (64%) decided to use cannabis based on their own experiences, with only 24% citing PCP advice.

**Table 1:** Sociodemographic characteristics of 275 medical cannabis patients at a Michigan medical cannabis dispensary

*Descriptive*	*Value*
**Gender**	
Men	38.5% (*n* = 106)
Women	59.6% (*n* = 164)
Other	0.4% (*n* = 1)
Missing	1.5% (*n* = 4)
**Age in years (*M*, SD, range)**	45, 16, 18–79
**Education**	
Grades 1–11	1.1% (*n* = 3)
High school graduate or GED	9.4% (*n* = 26)
Some college, no degree	23.2% (*n* = 64)
Associate’s degree	12.7% (*n* = 35)
Bachelor’s degree	30.2% (*n* = 83)
Master’s degree	13.8% (*n* = 38)
Doctorate (PhD, MD, etc.)	5.4% (*n* = 15)
Other	2.5% (*n* = 7)
Missing	1.5% (*n* = 4)
**Currently a student**	10.5% (*n* = 29)
**Races/ethnicities (inclusive)**	
White/European American	88% (*n* = 242)
Black/African American	5.1% (*n* = 14)
Hispanic/Latino	4.4% (*n* = 12)
American Indian or Alaska Native	2.2% (*n* = 6)
Asian	0.4% (*n* = 1)

*M* mean, *SD* standard deviation, *GED* general equivalency exam. Note: for race/ethnicities, participants could select all categories that applied. There were no participants who were Hawaiian/Pacific Islander

### Disclosure of medical cannabis use to PCP

All participants indicated having a PCP, and 81% reported that their PCP knew they used medical cannabis. Of the latter, 93% informed their PCP, 2% were asked about medical cannabis use by their PCP, 2% reported that their PCP recommended they use cannabis, and 3% said their PCPs found out another way (e.g., urine screen). Of participants whose PCP knew they used medical cannabis, 38% reported that their PCP had not always known. Of those whose PCP did not know they used medical cannabis, 81% (*n* = 39) did not tell their PCP for reasons including perceived stigma, PCPs having unfavorable attitudes towards medical cannabis, not wanting cannabis on their health records, fear of losing licensure or employment, and fear of being denied insurance or medical care.

### Cannabis licensure

Although 78% of participants saw their PCP for the health issue that they used cannabis to treat, only 14% obtained medical cannabis authorization directly from their PCP, compared to physicians identified through internet searches (40%), friends or family members (37%), dispensaries (9%), other medical personnel (5%), newspaper (4%), and other (3%) methods. Older participants were more likely to have their cards authorized by their PCP, *r*(270) = .180, *p* < .001. Only four participants (1.7%) whose medical cannabis was authorized by another physician were referred to that physician by their PCP. Most (74%) participants reported that the physician authorizing their medical cannabis license had no further involvement in their current healthcare, only 24% ever saw them again, and 16% reported that the authorizing physician was currently involved in their health care. Only 9% of participants reported that their PCP was in contact with the authorizing physician. Most participants visited one physician to authorize their license, although some visited as many as six (Table 2).

**Table 2:** Characteristics of and perceptions towards primary care providers of 275 Michigan medical cannabis patients

*Variables and responses*	*Value*
**Number of physicians visited to obtain medical cannabis authorization**	
1	75.3% (*n* = 207)
2	18.9% (*n* = 52)
3	2.9% (*n* = 8)
4	0.7% (*n* = 2)
5	0.4% (*n* = 1)
6	0.4% (*n* = 1)
Missing	1.5% (*n* = 4)
**Primary care provider’s knowledge about medical cannabis**	
Poor	32.4% (*n* = 89)
Fair	27.3% (*n* = 75)
Good	22.2% (*n* = 61)
Very good	12.4% (*n* = 34)
Excellent	4.4% (*n* = 12)
Missing	1.5% (*n* = 4)
**Confidence in primary care provider’s ability to integrate medical cannabis into treatment**	
Not at all confident	46.2% (*n* = 127)
Somewhat confident	16.7% (*n* = 46)
Moderately confident	16.7% (*n* = 46)
Very confident	10.2% (*n* = 28)
Completely confident	8.7% (*n* = 24)
Missing	1.5% (*n* = 4)
**Primary care provider’s level of support for medical cannabis**	
Not at all supportive	25.1% (*n* = 69)
Somewhat supportive	28.4% (*n* = 78)
Moderately supportive	21.1% (*n* = 58)
Very supportive	12.4% (*n* = 34)
Completely supportive	11.6% (*n* = 32)
Missing	1.5% (*n* = 4)

The majority of participants felt that their PCPs knowledge of medical cannabis was poor were not confident in their PCP’s ability to integrate medical cannabis into treatment, and viewed the PCP as not at all or somewhat supportive of medical cannabis

*PCP* primary care provider

### Perceptions of PCP knowledge of medical cannabis

Participants reported generally low confidence in their PCP’s ability to integrate medical cannabis into treatment, perceived their PCP’s knowledge about medical cannabis as poor or fair, and thought that PCPs were not at all, somewhat, or moderately supportive of medical cannabis (Table 2). Compared to participants whose PCP did not know about their medical cannabis use, those whose PCP did know rated their PCP as more knowledgeable about medical cannabis *t*(248) = 3.87, *p* < .001, *d* = .62, were more confident in their PCP’s ability to integrate medical cannabis into their treatment, *t*(248) = 4.34, *p* < .001, *d* = .70, and perceived their PCP as more supportive of medical cannabis, *t*(248) = 6.73, *p* < .001, *d* = 1.08. Similar trends were found for participants who delayed telling their PCP (*d* = 0.33–0.60, all *p*’s < .021) compared to those whose PCP always knew about their cannabis use. Non-Whites rated their PCP as more knowledgeable about medical cannabis, *t*(269) = 2.51, *p =* .013, *d* = 0.49, and more able to integrate it into their treatment, *t*(269) = 2.04, *p* = .042, *d* = 0.40, but not more supportive of medical cannabis, *t*(269) = 1.14, *p* = .254, *d* = 0.23. However, the sample size for non-whites was quite small (*n* = 33).

### Substitution for pharmaceuticals—with and without PCP knowledge or authorization

Overall, 86% (*n* = 235) reported using pharmaceutical drugs, 82% (*n* = 194) of whom reported reducing or stopping use of a drug because of their medical cannabis use. Substitution rates ranged from 36% for antihistamines to 88% for sedatives (Table 3). Most (87% on average) reported that their substitution decision was based on their own experiences compared with 18% citing PCP advice. Only 31% (*n* = 60) immediately reported substitution to their PCP, while 69% (*n* = 134) reported some gap in their PCP’s knowledge of their substitution, with 44% reporting that their PCP was not currently aware of this substitution.

**Table 3:** Cannabis substitution for pharmaceuticals among 275 Michigan medical cannabis patients, influences for substitution, and primary care provider knowledge of substitution

			Influences for substitution (%)				
Substance type	Use *n* (%)	Substitution (%)	Experiences	Healthcare provider	Dispensary	Individuals	Other
Amphetamines	52 (19%)	75	89	11	0	3	14
Anticonvulsants	30 (13%)	70	87	22	4	8	4
Antidepressants	141 (51%)	53	93	22	7	6	6
Antiemetics	33, 12%	79	87	13	4	0	4
Antihistamines	94, 34%	36	100	3	0	3	6
Antipsychotics	19 (7%)	78	71	43	0	7	0
Anxiolytics or benzodiazepines	64 (23%)	73	87	34	4	8	8
Muscle relaxers	107 (39%)	82	88	15	3	5	11
Non-opioid pain relievers	93 (34%)	76	97	10	4	6	12
Nonsteroidal anti-inflammatories	125 (46%)	62	92	14	3	4	9
Opioids	102 (37%)	86	88	28	11	9	12
Non-amphetamine stimulants	19 (7%)	50	89	11	0	11	22
Prescription cannabinoids	5 (2%)	75	75	0	0	25	25
Sedatives	34 (12%)	88	93	25	7	4	14
Sleep aids	79 (29%)	83	95	15	6	8	5
Steroids	44 (16%)	64	55	18	2	0	2

*N* = 275 participants total, 192 of whom reported any substitution. Note: all values are proportions of relevant groups. Use indicates proportion of participants who have used a substance. Substitution indicates proportion of participants using a substance who have reduced or stopped their use because of medical cannabis. Influences for substitution include participant’s own experimentation and experiences, advice from primary health/medical care provider, advice from my medical marijuana caregiver/dispensary, advice from other individual(s), and other source of information

*PCP* primary care provider

Compared to participants whose PCP always knew about their substitution, those who reported delayed PCP substitution knowledge rated their PCP as less knowledgeable about medical cannabis *t*(192) = 3.71, *p* < .001, *d* = .57, were less confident in their PCP’s ability to integrate medical cannabis into their treatment *t*(192) = 4.50, *p* < .001, *d* = .70, and perceived their PCP as less supportive of medical cannabis *t*(192) = 3.18, *p* = .002, *d* = .49. Identical trends were found for participants whose PCP was not aware of their pharmaceutical substitution (*d*’s = 0.54–0.60, all *p*’s < .001) compared to participants whose PCP currently knew about their substitution.

**Fig. 1:**
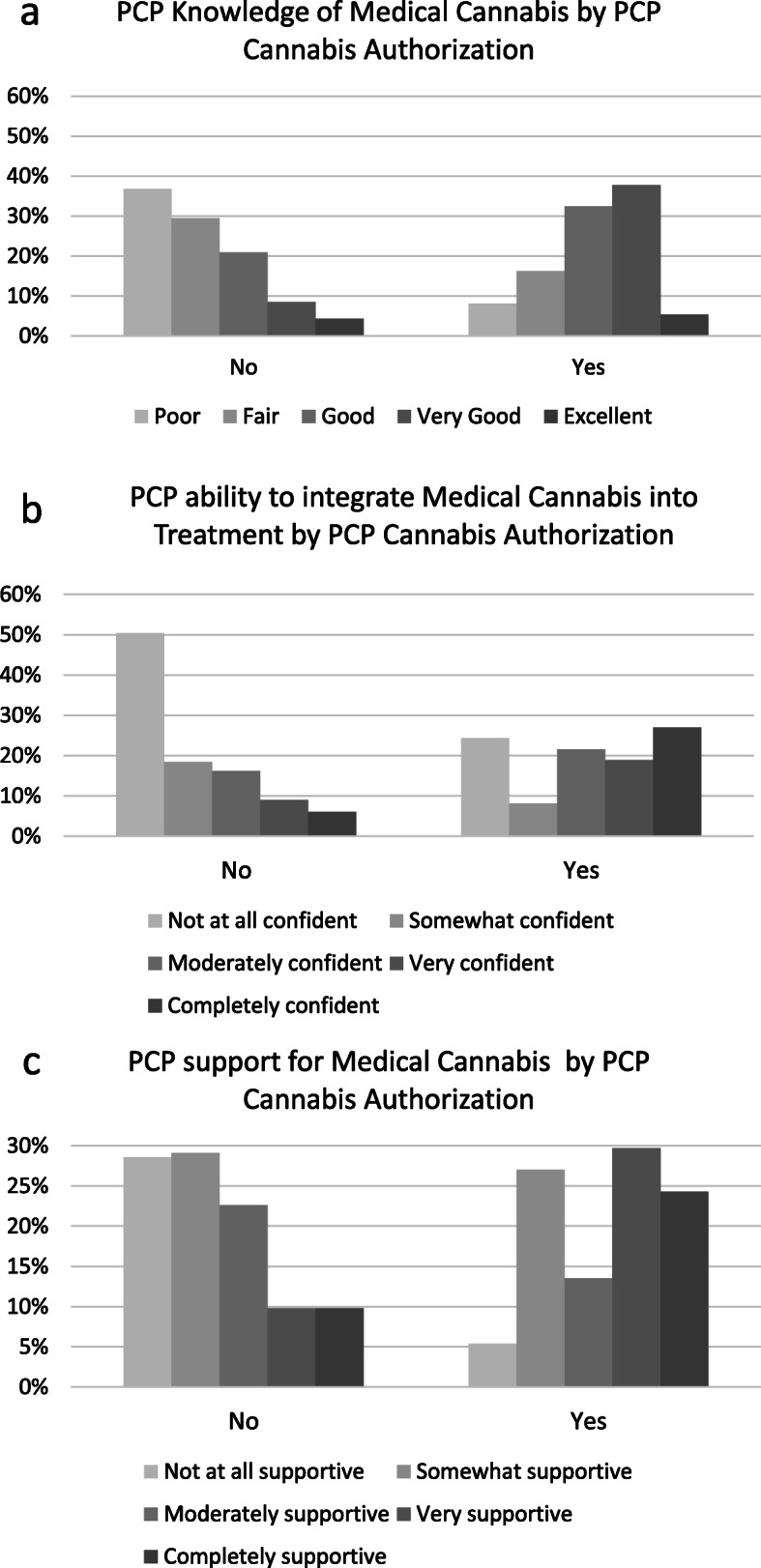
Patient perceptions by whether primary care provider (PCP) authorized cannabis licensure

Conversely, participants whose PCP authorized their medical cannabis card (*n* = 37) rated their PCP as more knowledgeable about medical cannabis *t*(269) = 5.14, *p* < .001, *d* = .91 were more confident in their PCP’s ability to integrate medical cannabis into their treatment *t*(269) = 5.00, *p* < .001, *d* = .88, and perceived their PCP as more supportive of medical cannabis *t*(269) = 4.33, *p* < .001, *d* = .77, than participants whose PCP had not authorized their medical cannabis card (Fig. 1). The distribution of PCP gaps in knowledge around participants substitutions were significantly different, *χ*^2^(1) = 4.35, *p* = .037, with more of those whose PCP had authorized their cannabis license reporting current PCP knowledge of this substitution. There was no difference in pharmaceutical substitution rates based on who had authorized participants’ medical cannabis cards, *χ*^2^(1) = 0.09, *p* = .760.

## Discussion

In this study, we show that although many participants reported substituting cannabis for medications, the majority did not have their medical cannabis license authorized by their PCP and reported substituting cannabis for medications based on their personal experiences rather than advice from their PCP. Further, many delayed telling their PCP about this medication substitution. Substitution patterns align with results from ecological (Bradford and Bradford 2016; Bradford and Bradford 2017; Bradford et al. 2018) and individual-level studies (Boehnke et al. 2016; Boehnke et al. 2019b; Lucas et al. 2016; Lucas and Walsh 2017; Lucas et al. 2019; Reiman et al. 2017; Piper et al. 2017; Corroon Jr. et al. 2017; Rod 2019) describing medication-sparing effects of medical cannabis legislation and use. To our knowledge, however, this is the first account of how substitution fits into mainstream medical care—or in this case, how far outside this context it occurs. This finding is consistent with our hypotheses and aligns with participant perceptions of minimal PCP knowledge of medical cannabis and low confidence in PCPs integrating cannabis into treatment. This substitution finding also raises safety concerns: although substituting cannabis for medications may be an appropriate harm reduction strategy in some cases (e.g., cannabis for opioids in the context of chronic pain) (Lucas 2017), doing so without PCP oversight may harm patients—e.g., disease recurrence if substituting for disease modifying drugs. Our findings highlight this latter point, as some participants reported substituting cannabis for stimulants, which are not used for any conditions (e.g., weight loss, narcolepsy) for which cannabis has known therapeutic value. These findings emphasize the need for education on cannabis so PCPs can provide appropriate counseling on safety and harm-reduction for patients who use medical cannabis.

Given potential legal repercussions for cannabis use due to its status as a Schedule I drug, it is unsurprising that medication substitution often occurs without PCP oversight and that many patients go to outside physicians to obtain cannabis licensure. Further, many PCPs may be uncomfortable recommending cannabis to patients given their lack of relevant education regarding medical cannabis use and cannabis legality (Kondrad and Reid 2013; Carlini et al. 2017). Indeed, a recent systematic review of healthcare provider attitudes towards medical cannabis confirmed that although healthcare professionals showed modest support for using medical cannabis in clinical practice, this support was tempered by “a lack of confidence, a lack of self-reported competence, and concerns for associated risks” (Gardiner et al. 2019). These concerns are supported by the lack of physician knowledge around cannabis’s legal status, demonstrated by a recent survey of physicians (*n* = 371) which reported only 34% knew that cannabis was a Schedule I drug, 68% knew it was federally illegal, and 65% could correctly identify the legality of cannabis in their state of residence (Takakuwa et al. 2020). In addition, among a survey of *n* = 494 family or internal medicine physicians in Washington State, the most common places for physicians to obtain medical cannabis-related information were patients, fellow providers, news media, and medical journals, rather than formal training programs (Carlini et al. 2017).

As shown by our findings and other reports in the scientific literature, however, conversations about medical cannabis between patients and their PCPs appear to result in better exchange of information that could keep patients safe. Indeed, adults ≥ 60 years old reported positive outcomes with medical cannabis and preferred to discuss their cannabis use with their healthcare provider (Bobitt et al. 2019). In a recent nationally representative survey, the 24% of respondents who indicated that healthcare providers were their most influential source of information were less likely to endorse incorrect beliefs about cannabis (e.g., cannabis is not at all addictive) (Ishida et al. 2020). However, cannabis’s illicit status may get in the way of building strong patient-PCP relationships, as some adults prefer to use cannabis illicitly because of concerns about their “name going on a list” or that their careers may be negatively impacted by disclosure of cannabis use (Lau et al. 2015).

### Implications

Our study highlights the need for better integration between medical cannabis and mainstream healthcare, including enhancing PCP education on cannabis, the endocannabinoid system, and the benefits, risks, and harms of cannabis in relevant therapeutic contexts. As medical cannabis policy allows cannabis to be used for many conditions for which there is no known therapeutic benefit (Boehnke et al. 2019a), education efforts should also focus on harm-reduction strategies aligned with current practical dosing guidance (e.g., slowly titrating doses and using multiple administration routes) (Savage et al. 2016; MacCallum and Russo 2018; Boehnke and Clauw 2019). Applying this guidance in clinical practice would give PCPs better tools to assess safety and effectiveness of currently available cannabinoid products and could quickly feed back into clinical practice. As numerous clinical trials are underway, consistently updating this practical guidance based on the most recent data is critical.

### Future directions

Future research should focus on developing actionable strategies to improve patient-PCP relationships and enhance shared decision-making in the context of medical cannabis and medication substitution. Examining and identifying the most challenging barriers to patient-PCP communication about cannabis could inform these studies. In addition, it would be worth investigating patient satisfaction with different medications they are currently taking to gauge which medication classes are the most likely targets for substitution with cannabis, as well as patient interest in and/or rationale for substituting. For example, while there are three currently approved medications for fibromyalgia, consumer surveys typically do not rank them as being very helpful for symptom management and frequently discontinue use (Hauser et al. 2012; Wolfe et al. 2013). We would thus expect there to be higher rates of substitution among people with fibromyalgia, a finding borne out by current observational studies of medical cannabis use (Sagy et al. 2019). Identifying similar clinical situations—especially those in which there is evidence of cannabis’ therapeutic value – could thus provide ideal research settings for piloting interventions focused on enhancing joint patient-PCP decision making.

### Limitations

Our study was limited in several ways. First, our results on medication substitutions and PCP attitudes are subject to recall bias and we do not have objective measures showing whether the reported substitution actually occurred. Second, our results reflect a mostly White population who obtained medical cannabis licenses, so they may not be generalizable to all people who use cannabis medically. Third, our results may be influenced by selection bias, as we do not know the total number of individuals who had the opportunity to take this survey nor do we know by which methods participants were most likely to be recruited (e.g., email, flyer). Fourth, our sampling was limited to a single dispensary in Michigan, which has had a medical cannabis law since 2008, as well as an adult use cannabis law during the sampling period. Michigan also does not have a strict regulatory process in place that mandates cannabis-specific training for physicians, either for those who write recommendations or broadly. Thus, the experiences of our study population may not translate to individuals in states with different medical cannabis infrastructure. Fifth, the perceptions of PCP knowledge and comfort around cannabis may inaccurately represent the reality of those specific care providers. However, given the widespread discomfort voiced by healthcare providers around cannabis, we believe this study adds an important facet to the scientific literature.

## Conclusion

While patients substitute cannabis for other medications, many do not disclose this substitution to their PCPs and perceptions of PCP expertise with cannabis and ability to integrate cannabis into medical care range widely. Similarly, although many medical cannabis patients tell their PCP about their use of medical cannabis, their license was typically authorized by an outside physician who had no current role in the patient’s healthcare. Our results show the poor integration between medical cannabis and mainstream healthcare, suggesting a need for improved physician education around appropriate cannabis use.

## Supplementary Information

**Additional file 1.**

## Data Availability

Data are available upon request.
