# Supplementary material for: Communication between healthcare providers and medical cannabis patients regarding referral and medication substitution
Source: J Cannabis Res. 2021 Jan 24;3:2. doi: 10.1186/s42238-021-00058-0 (PMC7831240; doi:10.1186/s42238-021-00058-0)
Supplement: Supplementary file 1 — Additional file 1. [file 42238_2021_58_MOESM1_ESM.docx]

Are you 18 years of age or older and have authorization for the use of medical marijuana/cannabis in the state where you live?

o Yes

o No

What is the primary condition that you use medical marijuana, in all its forms, to help treat? **(Check only one)**

o AIDS/HIV

o Anxiety or panic attacks

o Appetite or anorexia

o Arthritis

o Autism

o Attention Deficit (ADD or ADHD)

o Back spasms or back problems other than pain

o Brain Injury

o Cancer/Leukemia

o Chronic pain

o Crohn's disease

o Chronic fatigue

o Depression or bipolar disorder

o Diabetes

o Eating disorder

o Endometriosis

o Epilepsy

o Fibromyalgia

o Gastrointestinal

o Glaucoma

o Headache or migraines

o Hepatitis

o Hypertension

o Irritable Bowel Syndrome

o Kidney problems

o Movement disorder

o Multiple Sclerosis

o Nausea

o Nonepileptic seizure disorder

o Obsessive-Compulsive Disorder

o Osteoporosis

o Polycystic ovary syndrome (PCOS)

o Post-traumatic stress disorder (PTSD)

o Sciatica

o Scoliosis

o Skin condition

o Sleep issues/Insomnia

o Stress

o Temporomandibular joint (TMJ) syndrome

o Wasting syndrome

o Other: ________________________________________________

*[Kruger DJ, Kruger JS. Medical Cannabis Users' Comparisons between Medical Cannabis and Mainstream Medicine. J Psychoactive Drugs. 2019;51(1):31-36.; Kruger DJ, Kruger JS, Collins RL. Cannabis Enthusiasts' Knowledge of Medical Treatment Effectiveness and Increased Risks from Cannabis Use. Am J Health Promot. 2020:890117119899218.]*

What made you decide to use marijuana for health or medical purposes? **(check all that apply)**

▢ My own experiences

▢ Advice from my primary health/medical care provider

▢ Advice from my medical marijuana caregiver/dispensary

▢ Advice from other individual(s): _____________________________________________

▢ Other source of information: ________________________________________________

*[Kruger DJ, Kruger JS. Medical Cannabis Users' Comparisons between Medical Cannabis and Mainstream Medicine. J Psychoactive Drugs. 2019;51(1):31-36.; Kruger DJ, Kruger JS, Collins RL. Cannabis Enthusiasts' Knowledge of Medical Treatment Effectiveness and Increased Risks from Cannabis Use. Am J Health Promot. 2020:890117119899218.]*

A **primary care provider** is a physician, nurse practitioner, or other medical professional allowed under state law to provide medical care and coordinate or help a patient access a range of health care services.

Does your primary care provider know that you use medical marijuana?

o Yes

o No

o Don't know

Are you seeing (or did you see) your primary care provider for the health issue that you use medical marijuana to help treat?

o Yes

o No

How did your primary care provider find out that you use medical marijuana?

o I told her/him.

o S/he asked me about it.

o Other: ________________________________________________

*[Display This Question: If: Does your primary care provider know that you use medical marijuana?” = Yes]*

Was there a time when your primary care provider did not know that you used medical marijuana?

o Yes

o No

*[Display This Question: If: Does your primary care provider know that you use medical marijuana?” = No]*

Did your primary care provider ever ask you about medical marijuana?

o Yes

o No

*[Display This Question: If: Does your primary care provider know that you use medical marijuana?” = No]*

Is there a reason why you did not tell your primary care provider about your medical marijuana use?

o Yes (please explain) ________________________________________________

o No

Did your primary care provider authorize your medical marijuana card?

o Yes

o No

How many different doctors did you have to visit in order to get your medical marijuana card?

o 1

o 2

o 3

o 4

o 5

o 6

o 7

o 8

o 9

o 10+

*[Display This Question: If: Did your primary care provider authorize your medical marijuana card? = No]*

Is your primary care provider in contact with the physician who issued your medical marijuana card?

o Yes

o No

*[Display This Question: If: Did your primary care provider authorize your medical marijuana card? = No]*

How did you find the physician who authorized your medical marijuana card?

o Referred by my primary care provider

o Referred by a friend or family member

o In a newspaper (Metrotimes, etc.)

o Internet search

o Other: ________________________________________________

*[Display This Question: If: Did your primary care provider authorize your medical marijuana card? = No]*

After you received your medical marijuana card, was the doctor who authorized it involved in your health or medical care?

o Yes

o No

How would you rate your primary care provider’s knowledge about medical marijuana?

o Poor

o Fair

o Good

o Very Good

o Excellent

How confident are you in your primary care provider’s ability to integrate medical marijuana into your treatment?

o Not at all confident

o Somewhat confident

o Moderately confident

o Very confident

o Completely confident

How supportive is your primary care provider towards medical marijuana?

o Not at all supportive

o Somewhat supportive

o Moderately supportive

o Very supportive

o Completely supportive

Have you ever used (including if you are currently using or taking) … **(check all that apply)**

▢ Alcohol

▢ Amphetamines

▢ Antihistamines

▢ Anticonvulsants (anti-epileptic drugs, anti-seizure drugs)

▢ Antidepressants

▢ Antiemetics (for nausea and vomiting)

▢ Antipsychotics

▢ Anxiolytics/benzodiazepines (anti-anxiety)

▢ E-cigarette/nicotine vape

▢ Muscle relaxers

▢ Non-Opioid pain relievers

▢ NSAID - Nonsteroidal anti-inflammatory

▢ Opioids

▢ Other stimulants (not amphetamines)

▢ Prescription cannabinoids - (Cesamet, Marinol, Sativex)

▢ Sedatives

▢ Sleep aids

▢ Steroids

▢ Synthetic cannabinoids - (K2, Spice, etc.)

▢ Tobacco

▢ Other pharmaceutical drug #1: ________________________________________________

▢ Other pharmaceutical drug #2: ________________________________________________

▢ Other pharmaceutical drug #3: ________________________________________________

▢ Other drug #1: ________________________________________________

▢ Other drug #2: ________________________________________________

▢ Other drug #3: ________________________________________________

*[Kruger DJ, Kruger JS. Medical Cannabis Users' Comparisons between Medical Cannabis and Mainstream Medicine. J Psychoactive Drugs. 2019;51(1):31-36.]*

*[For each option selected in the item above, display following three items:]*

Have you reduced your use of or stopped using **[drug]** because of medical marijuana?

o Yes

o No

o Don't know

*[Boehnke KF, Scott JR, Litinas E, Sisley S, Williams DA, Clauw DJ. Pills to Pot: Observational Analyses of Cannabis Substitution Among Medical Cannabis Users with Chronic Pain. J Pain. 2019;20(7):830-841.]*

What made you decide to reduce or stop your use of **[drug]**? (check all that apply)

▢ My own experimentation and experiences

▢ Advice from my primary health/medical care provider

▢ Advice from my medical marijuana caregiver/dispensary

▢ Advice from other individual(s): _____________________________________________

▢ Other source of information ________________________________________________

*[Kruger DJ, Kruger JS. Medical Cannabis Users' Comparisons between Medical Cannabis and Mainstream Medicine. J Psychoactive Drugs. 2019;51(1):31-36.]*

Did your primary care provider know that you reduced or stopped your use of **[drug]** because of medical marijuana?

o Yes, immediately

o Yes, but not immediately

o No

**Demographics**

What is your age in years? ____________________

You are:

o Female

o Male

o Other (please describe) ________________________________________________

What races/ethnicities do you identify with? (Please check all that apply)

▢ American Indian or Alaska Native

▢ Asian

▢ Black/African American

▢ Hispanic/Latino

▢ Native Hawaiian/Pacific Islander

▢ White

▢ Other ________________________________________________

Are you a currently a student?

o Yes

o No

What is the highest grade or degree you have completed?

o Grades 1-8: ________________________________________________

o Grades 9-11 ________________________________________________

o High school graduate or GED

o Some college, no degree

o Associate's Degree

o Bachelor’s Degree

o Master’s Degree

o Doctorate (PhD, MD, etc.)

o Other: ________________________________________________
